# Production of Bioproducts from Wastewater Treatment Using the Microalga *Neochloris oleoabundans*

**DOI:** 10.1002/elsc.70032

**Published:** 2025-07-03

**Authors:** Fahd Mnasser, Mª Lourdes Martínez‐Cartas, Sebastián Sánchez

**Affiliations:** ^1^ Department of Chemical, Environmental and Materials Engineering Higher Polytechnical School of University of Jaén Linares Spain; ^2^ University Institute of Research in Olive Grove and Olive Oils University of Jaén Mengíbar Spain

**Keywords:** bioproducts, circular bioeconomy, *Neochloris oleoabundans*, olive and oil washing wastewater, urban wastewater

## Abstract

Olive mill wastewater (OMW) poses an environmental risk due to its high chemical oxygen demand (COD). It is also rich in nutrients and organic carbon and has been used in this study together with urban wastewater (UWW) as part of the *Neochloris oleoabundans* culture medium in different proportions to analyze the results in the change of proportion in the mixtures, looking for the highest level of wastewater utilization. The objective was to determine both the growth capacity of the microorganism in this medium for subsequent production of bioproducts (such as pigments, crude protein, or lipids) from the generated microalgal biomass, as well as the rate of removal of compounds present in wastewater after treatment, which enables it to be discharged into public canals and used for irrigation. The bioprocesses which were developed in batch photobioreactors of 1 dm^3^, showed the highest values of maximum specific growth rate and volumetric biomass productivity 0.082 h^−1^ and 3 mg dm^−3^ h^−1^, respectively. The maximum percentage of crude proteins (49.5%), lipids (51.0%), and carbohydrates (56.0%) we obtained in the final biomass. In this regard, the biggest percentages of organic load removal in the treatment with *N. oleoabundans* were 65.66% for total phenolic compounds and 93.7% for COD. In addition, the potential content of salts such as nitrates, nitrites, and orthophosphate decreased for 97.6%, 94.3%, and 69.2%, respectively. Values that show the suitability of *N. oleoabundans* for the recovery of bioproducts and the reduction of the pollutant load of wastewater for its reuse in agriculture, increasing the circular bioeconomy in the olive sector.

**Summary:**

- In a region with the highest olive oil production in the world, the high generation of waste from the olive oil industry requires the implementation of solutions to valorize and minimize the waste generated. The treatment of olive mill wastewater, used as a culture medium providing nutrients for the development of microalgae, is a promising procedure.
- In this study, the microalgae *Neochloris oleoabundans* has been used to treat olive oil mill wastewater, which has allowed high yields to be obtained in the production of biomass that can be transformed into bioproducts such as carbohydrates, proteins, and lipids. In addition, a high yield is obtained in the elimination of components that can be harmful, which is relevant for the reuse of water for irrigation or for discharge into natural watercourses.
- The experimental results of this work have also considered the ability of *N. oleoabundans* to purify urban wastewater from secondary wastewater treatment.

## 1 Introduction

The olive oil industry is economically one of the most important in the agri‐food sector of Mediterranean countries [1]. As the main ingredient in the traditional Mediterranean diet, olive oils have positive effects on human health. The health benefits attributed to this product are mainly due to its richness in antioxidants [2]. However, the olive oil production process generates high amounts of solid and liquid wastes. The treatment of oil mill wastewater (OMW) is one of the main environmental problems faced by the Mediterranean countries, where Spain is the leading olive oil producer [3]. The water consumption in the continuous centrifugation system, using a decanter of two outlets (90% of olive oil production in Spain is carried out using this system), what represents about 10–18 dm^3^ of water per 100 kg of olives. During olive wash operation, the consumption is about 10–12 dm^3^ of water per 100 kg of olives. In the vertical centrifuge, about 5–6 dm^3^ of water is added per 100 kg of olives. In this process, from 100 kg olives, about 20 kg of oil are obtained, and as by‐products 77.3 kg of wet olive pomace (WOP) containing 61.5% water and 5–6 dm^3^ of oil mill wastewater [4].

In general, OMW is characterized by a very high organic load, due to high levels of phenolic compounds and sugars, and has minimal levels of nitrogenous compounds, low pH, low dissolved oxygen (DO), and dark coloration [5, 6]. The organic fraction, in addition to phenolic compounds and sugars, also includes polyalcohols [7], tannins and a high content of lipids and other substances that are difficult to degrade. Phenolic compounds, present in the olive stone and pulp, tend to be more soluble in the aqueous phase than in the oil. Furthermore, these wastewaters present high values of biological oxygen demand (BOD_5_) and chemical oxygen demand (COD) [8]. Moreover, high concentration of dark‐colored polyphenols can discolor streams and rivers, high concentration of reduced sugars can stimulate microbial respiration, low DO content and high phosphorus content can lead to eutrophication [6]. The high content of polyphenols and fatty acids can inhibit the growth of microorganisms and stop conventional secondary and anaerobic treatments in municipal treatment plants [9]. The potential of OMW for volatile fatty acid (VFA) and sugar production has also been studied, considering it composition [10]. In other cases, the purification of OMW by oxidation processes has been studied [11, 12], producing water that can be used for irrigation or discharged directly into the municipal wastewater system for further tertiary treatment indicating, as a general rule, that olive oil mill wastewater from olive washing does not exceed the permissible limits for reuse in irrigation. In fact, Spanish environmental standards consider OMW removal practices, to be used in the irrigation of agricultural land [13], assuming the water quality standards for agriculture of the FAO [14] and the EU [15], which establish as limits for the water for irrigation.

Regarding to urban wastewater (UWW), human activities have contributed to a substantial degradation of water quality in aquatic ecosystems around the world. Currently, large amounts of wastewater are produced every year and wastewater emissions continue to increase []. UWW is a mixture of wastewater from different sources such as water from domestic, commercial, and industrial activities [12]. The amount generated as well as the physico‐chemical properties depend on the standard of living, behavior and lifestyle of the inhabitants of the regions where, it is generated. In addition, the design of the wastewater system significantly influences the composition of this water UWW [16] contains organic and inorganic compounds, and its physicochemical composition is highly variable. Thus, the potential of microalgae to treat industrial and municipal wastewater, due to their high nutrient content, has been studied [17]. In most cases, the composition of wastewater alone is not optimal for microalgal biomass growth. However, blending different wastewater sources can be an effective strategy for optimizing culture conditions. The combination of OMW and UWW has been found to enhance the growth of *Scenedesmus obliquus* [18], as well as the removal of pollutants and the recovery of bioproducts [19].

This work proposes the use of olive washing wastewater (OLW) and olive pomace wastewater (OIW) as separate fractions, which represents a reduction in the volume of olive mill wastewater (OMW) as a total separated liquid fraction in olive oil production both in a mill with a 3‐outlet decanter and in a plant with a 2‐outlet decanter [20]. The influence of both types of water, separately, will be determined, which will maximize the volume of water used to a greater extent than when both types of water are treated together, taking into account their different physico‐chemical characteristics. This combination has advantages such as counteracting the inhibitory effect of OMW on the microorganism, [1].

Furthermore, microalgae are able to efficiently produce primary metabolites such as proteins and carbohydrates [21], lipids [22] and pigments [23]. In addition, microalgae could also be conveniently used for the bioremediation of contaminated wetlands [24, 25]. Microalgae have gained increasing attention as a resource for third‐generation biodiesel production [26] due to their high specific growth rates [27] and high lipid/fatty acid productivity [28] and for biofuel production considering their carbohydrates composition [29].

*Neochloris oleoabundans* is a terrestrial oleaginous microalgae species of the *Chlorophyta* family that can be cultivated in both fresh and salt water. It is characterized by being a fast‐growing triglyceride‐producing microalgae [30] that can accumulate up to 50% of lipids on a dry biomass basis [27, 31]. Therefore, it is interesting to research the potential of this species for the integrated treatment of oil mill effluents enriched with secondary municipal wastewater.

This study focuses on the growth of *N. oleoabundans* in OMW based culture medium with the dual objective of treating OMW and providing a low‐cost substrate for the production of microalgae biomass that could be used for biofuel production. For this purpose, two different types of OMW (OLW and OIW) with high and low initial inoculum, respectively, and with different proportions of double distilled water enriched with UWW from secondary treatment was used for the formation of culture medium. The growth kinetics, the biochemical composition of the final biomass, and the final quality of the treated water were evaluated. Oil mill wastewater combined with UWW from secondary treatment will be used as a culture medium to provide the substrate required for microalgae growth and development. The aim is to minimize the contaminating potential of the water for its reuse as irrigation water and to reduce its high pollutant content of certain metals such as (Na, Mg, B, and others) and ions such as nitrites, nitrates, or orthophosphates. At the same time, the microorganisms that are developed during the water purification process through various bioprocesses allow the recovery of bioproducts that can have added value and could be used to produce biofuels, thus contributing to improving the environmental sustainability of the sector by implementing the circular economy in the olive oil industry [32].

## 2 Materials and Methods

### 2.1 Microorganism Strain and Culture Conditions

In this study, we worked with the microalga *N. oleoabundans* UTEX 1185 from the Algae Culture Collection of the University of Texas, cultured in Rodríguez‐Lopez (R‐L) medium at 50% [33] in 250 mL Erlenmeyer flasks maintained at 25°C in a shaker under the following conditions. The cultures were illuminated with an initial illumination intensity 359 µE m−^2^ s−^1^, 12 h/day and shaken at 200 rpm, considering that excessive agitation can damage cells [34, 35]. Original stock cultures of *N. oleoabundans* were maintained in R‐L medium with the following chemical composition: KNO_3_ (10.11 g dm−^3^), Na_2_HPO_4_·12H_2_O (16.31 g dm−^3^), NaH_2_PO_4_·2H_2_O (0.78 g dm−^3^), MgSO_4_·7H_2_O (24.65 g dm^−3^), CaCl_2_·2H_2_O (1.47 g dm−^3^), FeSO_4_·7H_2_O (7.00 g dm−^3^), EDTA (9.30 g dm−^3^), MnSO_4·_H_2_O (0.17 g dm−^3^), ZnSO_4_·7H_2_O (0.29 g dm−^3^), CuSO_4_·5H_2_O (0.25 g dm−^3^), H_3_BO_3_ (0.061 g dm−^3^), and (NH_4_)_6_Mo_7_O_24_·4H_2_O (0.013 g dm−^3^).

### 2.2 SEM of *Neochloris oleoabundans*

**FIGURE 1:**
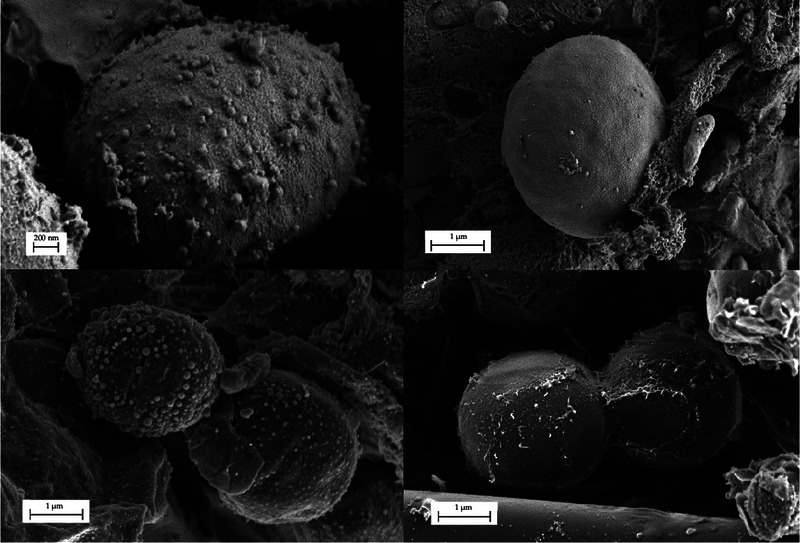
Scanning electron microscopy images of *Neochloris oleoabundans*.

Scanning electron microscopy (SEM) images of *N. oleoabundans* were obtained for the microalgae in initial conditions, prior to the development of the bioprocesses. Figure 1 shows different images of the initial state of the microorganism, using an electron microscopy (Carl ZEISS, model Merlin).

### 2.3 Characterization of Wastewater

Three types of wastewater were used: Olive oil washing wastewater (OIW), OLW, which comes from olive cultivation and is collected at the San Esteban de Mancha Real oil mill (Jaen), and UWW, which comes from the secondary treatment of the wastewater treatment plant (WWTP) in Mengíbar (Jaen). The olive OIW was collected in the decanter of the olive mill, and the OLW was compiled in the hopper; both were taken before these fractions were mixed by integrating the fraction corresponding to the OMW. For each of the experiments developed, the percentage of these different types of UWW was varied in a range of 0%–50%.

The wastewater used was characterized for pH, electrical conductivity (EC), DO, turbidity, total solids, organics, phenolic compounds, lipids, ash, DO, BOD_5_, COD, total phosphorus, nitrates, nitrites, sulfate, and phenolic compounds.

Total solids and ash were determined by drying and calcination of the samples in an oven at a temperature of 105 ± 1°C and in an oven at 575 ± 25°C, respectively. The difference between the two parameters corresponds to the percentage of organic matter. DO was measured directly with an oximeter (HANNA instruments optic/H1764113), and BOD_5_ was determined as the difference between the DO in the sample before and after 5 days in hermetically sealed bottles at 20°C in incubator Medilow. The COD was determined spectrophotometrically, using the Merck Spectroquant 1.14541.0001 method, based on dichromate oxidation. The conductivity of the effluent was measured by taking a 10 cm^3^ sample and using a previously calibrated conductivity meter (Cobra 4, Mobile‐Link). The turbidity measurement was carried out using a TN3024 turbidimeter in which a 10 cm^3^ sample of the effluent was taken. The pH was measured using a previously calibrated pHmeter CRISON.

The characterization of wastewater before and after the process was made using spectrophotometric methods for the ions: Nitrates reacting with 2,6‐dimethylphenol to form a nitro‐aromatic derivative detectable at 338 nm in sulfuric and phosphoric media (Merck, Spectroquant 1.14563.0001). Nitrites that react in an acid medium with sulfanilic acid to form a diazonium salt (Merck, Spectroquant 1.14547.0001). Orthophosphates form complexes with acid molybdate in acidic media, which, in the presence of ascorbic acid, are reduced to phosphomolybdenum blue (Merck, Spectroquant 1.14729.0001). Sulfates react with barium ions to form a sparingly soluble barium sulfate precipitate, which allows photometric measurement (Merck, Spectroquant 1.14548.0001).

Phenolic compounds were also determined using the Merck, Spectroquant 1.14551.0001 method. On the other hand, ions as magnesium, sodium, potassium, boron and copper, were measured using an ICP‐MS spectrometer Agilent 7900.

### 2.4 Bioprocesses for Olive Oil Mill and Urban Wastewater Treatment

A pretreatment was necessary to apply; by separating suspended solids from the water used, the thickening of the membranes can be reduced, and the effectiveness of OMW can be increased. Since the centrifuge is only used in the olive oil production cycle, it can also be used economically in the treatment of waste and is therefore preferred by other techniques such as microfiltration. In addition, the simplicity of the centrifugation process and the possibility of using a unique mechanical process (without chemical modifications) are an advantage of this technique over others.

Centrifugation as a pretreatment step not only achieves higher fluxes of centrifugated liquid, but also makes membrane cleaning easier and faster, thus improving the overall efficiency of OMW [36]. In addition, microfiltration through a membrane with a pore size of 0.45 µm was used to reduce the microbial (sterilization) and organic load of the OMW [37].

The culture of microalgae was made using batch glass photobioreactors type stirred tank at laboratory scale, under sterile conditions with front lighting, and a working volume of 1 dm^3^ per reactor [38]. In the installation three bioprocess were developed simultaneously.

The conditions of the different bioprocesses (BP) developed are shown in Table 1. In each photobiorreactor, the initial conditions were: pH = 7, % inoculum = 5, Agitation (12 h/day) = 200 rpm, aeration (12 h/day) = 0.018 m^3^/h−^1^ and light intensity (12 h/day) of 359 (µE m−^2^ s−^1^). The concentration of the microalgae inoculum varied between 0.3 and 1.1 g dm−^3^ and the proportions of wastewater in culture medium were changed for each experiment.

**TABLE 1:** Different proportions each type of wastewater, Rodríguez‐Lopez medium (R‐L) and bidistilled water (BDW) in each experiment.

	High inoculum concentration (1.1 g dm^−3^)					Low inoculum concentration (0.3 g dm^−3^)				
Exp	OIW^a^%	OLW^b^ %	UWW^c^ %	R‐L %	BDW %	OIW^a^%	OLW^b^ %	UWW^c^ %	R‐L %	BDW %
BP1	50	0	0	0	50					
BP2						50	0	0	0	50
BP3	0	50	0	0	50					
BP4						0	50	0	0	50
BP5	0	0	50	0	50					
BP6						0	0	50	0	50
BP7	50	10	10	0	30					
BP8	10	50	10	0	30					
BP9	10	10	50	0	30					
BP10	10	10	30	0	50					
BP11						10	10	30	0	50
BP12	10	10	10	50	20					
BP13						10	10	10	50	20
BP14	0	0	0	50	50					

^a^ OIW: Oil washing wastewater.

^b^ OLW: Olive washing wastewater.

^c^ UWW: Urban wastewater with secondary treatment.

Bioprocesses BP12, BP13, and BP14 have been carried out with a 50% of R‐L medium, serving as a benchmark of the better conditions with respect to the composition of the medium.

All materials and media used for the shake culture as well as for the development of the mentioned experiments were sterilized in an autoclave (SYSTEC VB‐40). The quantification of cell growth was performed using two kinetic parameters (maximum specific growth rate and biomass volumetric productivity). The cultures are maintained for the duration of the microorganism's growth phase a period of 15–25 days.

### 2.5 Characterization of *Neochloris oleoabundans*

#### 2.5.1 Microalgal Growth Measurement

**FIGURE 2:**
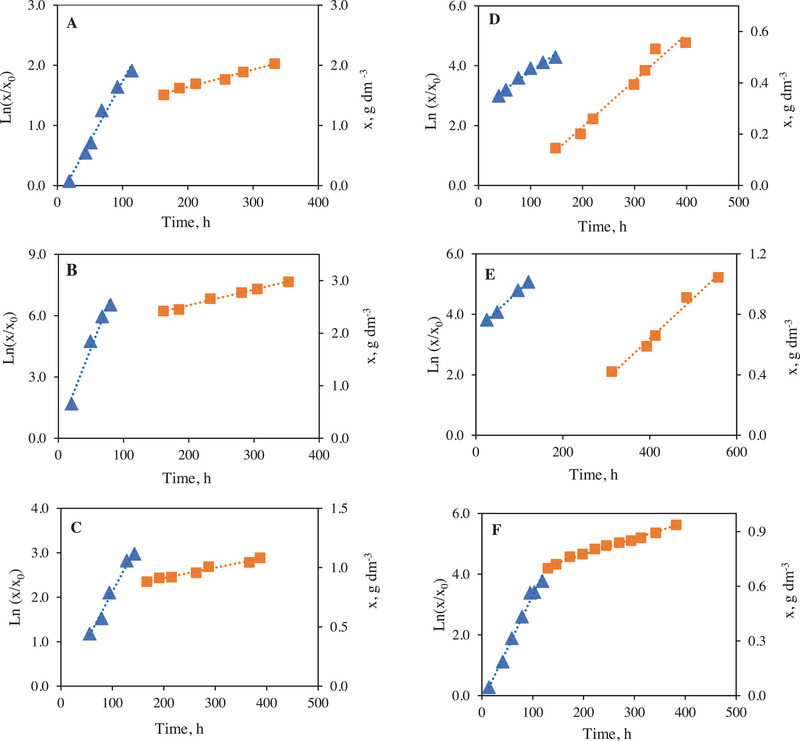
Growth curves of *Neochloris oleoabundans* cultivated in photobioreactor. (1) ln(x/x_0_) (▴). (2) Biomass, *x* (g/dm^3^) (◼). (A: BP14, B: BP12, C: BP1, D: BP3, E: BP 5, F: BP10).

The biomass concentration (x, g dm−^3^) was monitored during the experiments (Figure 2). For this purpose, a volume of 5 cm^3^ of the microalgae suspension was centrifuged at 4000 rpm for 10 min to allow the separation of biomass and culture medium. The biomass was then washed three times with double distilled water. The absorbance of the cell suspension was measured at 550 nm using a UV‐VIS spectrophotometer Biochrom Libra S60. In addition, pH, EC, DO and turbidity were measured in each sample taken.

#### 2.5.2 Pigments

Chlorophyll content was determined using a photocolorimetric method after extraction with 90% acetone [39]. In addition, carotenoid concentrations were quantified according to the method described in [40]. In order to calculate the amount of chlorophyll, it was necessary to measure the absorption at the wavelengths 647 and 664 nm spectrophotometrically. To estimate the concentration of carotenes, the absorbance was measured at a wavelength of 480 nm.

#### 2.5.3 Proteins, Total Lipids, and Carbohydrates

**FIGURE 3:**
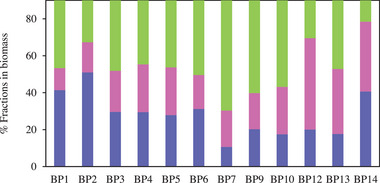
Biochemical composition of the dried biomass at the end of the (**◼** % Lipids, **◼** % Proteins, **◼** % Carbohydrates).

**FIGURE 4:**
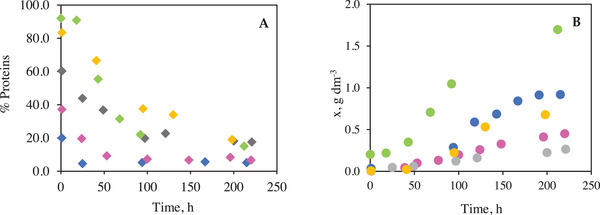
Evolution of proteins concentration cell relative to biomass concentration and time with (A: % Proteins vs. Time) and (B: Biomass concentration vs. Time). Where ⬥ is % Protein in BP1, ⬥ % Protein in BP3,4 ⬥ % Protein in BP5, ⬥; % Protein in BP10 and ● Biomass with BP1, ● Biomass in BP 3, ● Biomass in BP 5, ● Biomass in BP 10, and ● Biomass in BP 14.

In relation to protein, two parameters have been determined: the percentage of crude protein determined at the end of the experiment, referred to as dry matter, whose values are shown in Figure 3, and the daily measurement during the growth phase, shown in Figure 4. At the end of each experiment, the crude protein content of the harvested biomass was determined from total nitrogen (% TN) following elemental analysis. The biomass was collected by centrifugation (5000 rpm, 60 min), washed three times with double‐distilled water, and dried at 40°C in a vacuum oven. Crude protein was then calculated using the conventional nitrogen‐to‐protein conversion factor [41], according to Equation (1):

(1) %Crudeprotein=%TN×6.25

The protein content of *N. oleoabundans* was monitored throughout the experimental period and characterized in detail at the end of cultivation. Daily variations in cellular protein were assessed using the colorimetric Bradford method (1976) [42], which is based on the binding of Coomassie Brilliant Blue dye to proteins and subsequent spectrophotometric measurement. To this end, 5 cm^3^ samples of the cell suspension were centrifuged, washed twice with double‐distilled water, and resuspended to the original volume. Protein extraction was achieved via ultrasonication for 30 min at full power using a Branson Sonifier 450 in an ice bath. From the homogenized extract, 1.6 cm^3^ was mixed with 0.4 cm^3^ of Bio‐Rad reagent, gently stirred, and allowed to react for 10 min. Absorbance was then measured at 595 nm using 1 cm path‐length glass cuvettes. A reagent blank was prepared with double‐distilled water.

In addition, the dry biomass was characterized for total lipids, determined by extraction of the biomass with chloroform‐methanol (3:1) for 12 h in a micro‐soxhlet extractor and the ash content (mineral matter) quantified by calcining 0.1 g of dried biomass at 575°C for 3 h in a Selecta muffle furnace. Caution was exercised in interpreting ash content due to the potential overestimation from residual salts, particularly in marine‐derived microalgae. Carbohydrate content was estimated considering that proteins, lipids, and carbohydrates account for approximately 90% of microalgal dry weight, while nucleic acids and ash represent minor fractions [38, 43, 44, 45]. Nucleic acids were assumed to contribute around 4% [46].

### 2.6 Characterization of Separated Liquid Fraction

The liquid fraction separated after each bioprocess was characterized by measuring the same parameters used in the characterization of the starting wastewater, such as pH, EC, DO, turbidity, organic matter, phenolic compounds, fatty matter, DO, total phosphorus, nitrates and nitrites, sulfate compounds, and phenols, described in Section 2.3.

## 3 Results and Discussion

### 3.1 Physical‐Chemical Characterization of Wastewater

Oil mill wastewater OMW (OIW and OLW) and UWW have a nutritional profile suitable for use as a microalgae culture medium, with carbon, nitrogen, and phosphorus sources being the most important elements, required for the growth of algal biomass. It is necessary to underline, the strong presence of high organic matter, especially in the case of the OIW, determined in terms of EC, turbidity, concentrations of phenols compounds TPC, BOD_5_ mg O_2_ dm−^3^ and COD, as well as phosphorus in the form of inorganic salts (orthophosphate: 18 mg dm−^3^) and a significant concentration of nitrogen derivatives like nitrate and nitrite. The values of these parameters for the three types of wastewater are shown in Table 2, noting the high BOD_5_ and COD of OMW relative to UWW and the differences in ion content.

**TABLE 2:** The composition of treated industrial olive oil wastewater and urban wastewater.

	Olive washing wastewater (OLW)	Oil washing wastewater (OIW)	Urban wastewater (UWW)
pH	6.51 ± 0.05	5.86 ± 0.20	6.89 ± 0.15
Dissolved oxygen (mg dm*^–^* ^3^)	3.50 ± 0.10	3.95 ± 0.10	3.58 ± 0.10
electrical conductivity (µS/cm*^–^* ^1^, 25°C)	1058.20 ± 0.50	1928 ± 0.90	545.7 ± 0.65
Turbidity (NTU)	110 ± 0.30	935 ± 0.50	8.72 ± 0.05
Total solids (%)	0.71 ± 0.00	1.23 ± 0.01	0.79 ± 0.00
Ashes (%)	0.36 ± 0.00	0.19 ± 0.00	0.73 ± 0.00
Fat matter (%)	1.34 ± 0.00	3.49 ± 0.00	0.48 ± 0.00
Volatile organic matter (%)	0.35 ± 0.00	1.05 ± 0.00	0.067 ± 0.001
BOD_5_ (mg O_2_ dm*^–^* ^3^)	1000.0 ± 0.80	43,000.0 ± 0.8	590.0 ± 0.5
COD (mg dm*^–^* ^3^)	1722.0 ± 0.10	10,250.0 ± 0.1	120.0 ± 0.0
Total phenolic compounds (mg dm*^–^* ^3^)	6.60 ± 0.05	41.40 ± 0.08	0.70 ± 0.15
NO_3_ *^–^* (mg dm*^–^* ^3^)	3.20 ± 0.1	42.00 ± 0.5	5.00 ± 0.10
NO_2_ *^–^* (mg dm*^–^* ^3^)	0.25 ± 0.05	13.70 ± 0.13	3.53 ± 0.08
SO_4_^2^ *^–^* (mg dm*^–^* ^3^)	100.0 ± 0.0	230.0 ± 0.0	400.0 ± 0.0
PO_4_^2^ *^–^* (mg dm*^–^* ^3^)	3.00 ± 0.05	18.00 ± 0.80	4.00 ± 0.05
B^3+^ (mg dm*^–^* ^3^)	0.16 ± 0.01	0.16 ± 0.00	101.79 ± 0.50
Mg^2+^ (mg dm*^–^* ^3^)	14.18 ± 0.76	42.85 ± 1.07	< 0.00
K^+^ (mg dm*^–^* ^3^)	89.92 ± 0.90	106.40 ± 0.50	82.37 ± 0.10
Cu ^2+^(mg dm*^–^* ^3^)	0.29 ± 0.00	0.32 ± 0.01	0.019 ± 0.005
Na^+^ (mg dm*^–^* ^3^)	16.61 ± 0.90	23.41 ± 0.90	52.28 ± 0.80

Although the effectiveness of centrifugation [41] and microfiltration [35] depends on the species of microalgae selected, its efficiency at laboratory scale has obtained recovery rates of around 80%–90% in relatively short times (between 2 and 5 min). Table 3 shows the effect of centrifugation and microfiltration operations over the turbidity of the three kinds of wastewater.

**TABLE 3:** Variation of turbidity after centrifugation and microfiltration.

Turbidity, NTU				
	Before centrifugation	After centrifugation	After centrifugation + microfiltration	% turbidity reduction
OIW	1145.00 ± 1.00	87.00 ± 0.50	68.00 ± 0.75	94.06
OLW	110.00 ± 0.50	25.00 ± 0.70	20.12 ± 0.30	81.71
UWW	7.12 ± 0.20	1.67 ± 0.10	0.10 ± 0.05	98.60

The design of an adequate treatment system for this wastewater requires a thorough knowledge of its composition. Initially, the objective has been to establish the physicochemical characterization of the different liquid effluents generated in olive mills by means of the continuous centrifugation process with a two‐outlet decanter. In addition, UWW from secondary or biological treatments will be characterized. This UWW will be used in conjunction with that from oil mills.

The composition of wastewater from olive mills depends on different parameters, including the type of crop, harvest season, climate, and extraction method [47]. Furthermore, this water composition is variable, in term of the presence of its ions, which can be explained by the different composition of the agricultural soil, the variety, the ripeness index of the fruit, the origin of the water used to wash the oils, and even the use of talc as an adjuvant to improve the extraction of oil from olive pastes [48].

The possible inhibitory and toxic effect of OMW is mainly due to the presence of high concentrations of phenolic compounds, residual fats, and other substances that do not come from the olive tree. Many phenolic compounds are inhibitors of microorganisms, animals, and plants. Other components can be persistent and bioaccumulative; in particular, chlorinated forms can be toxic to human health [49]. The profile of phenolic compounds in oil wash water depends on several factors: variety, climatic conditions, fruit ripening, and olive extraction process [50]. Moreover, wastewater contains a large amount of organic matter that can be very valuable in the use of culture medium [51], and the issue resides in its ever‐changing composition [11, 12]. Martínez‐Nieto et al. [11] observed a greater concentration of phenolic compounds, sulfates, and iron in OIW compared to that of OLW.

Mineral forms of nitrogen assimilated by microalgae are nitrates (NO_3_^−^), nitrites (NO_2_^−^), and ammonium (NH_4_^+^). In Table 2, the OIW holds a very high concentration of nitrates and nitrites compared to those of OLW and UWW can be seen. This divergence between OIW and OLW can grow 13 times larger or more in the case of nitrates and almost 55 times in the case of nitrite. Consequently, the high consumption of these ions in the exponential phase could be explained, considering that the bioprocesses carried out with a major percentage of OIW registered a maximum growth concentration. Killberg‐Thoreson et al. [52] showed that organic sources served as nitrogen substrate for microalgae.

Microalgae are capable of assimilating phosphate for several uses. Algal growth incorporates phosphorus into constituents such as phospholipids, nucleotides, and nucleic acids, but algae are also capable of storing phosphorus in the biomass in the form of polyphosphates [53]. The phosphate content of OIW is 6 times higher than that of OLW and 4.5 times larger than that of UWW.

The composition of the culture medium is a paramount factor during the production of biomass, being well known the relationship between nutrient concentration and biomass composition [41]. The culture medium has an effect on the specific growth rate and the maximum level of biomass production. The deficiency of an essential nutrient in the culture medium causes an adaptation of the metabolism of the microalgae in response to new external conditions. In general, changes in the culture medium result in a variation in the biochemical composition of the biomass, for the most part: proteins, lipids, carbohydrates, and pigments. Additionally, several physical and chemical parameters can affect the growth of algae, involving light radiation, temperature, CO_2_, pH, agitation, aeration, and salinity.

### 3.2 Elemental Composition of *Neochloris oleoabundans*

Regarding the initial elemental composition of microalgae [54], the values of the biomass harvested at the end of each experiment are shown in Table 4. The results ranged from (51.18%–32.33%) for carbon (7.91%–1.91%) for nitrogen, (7.48%–5.12%) for hydrogen, and (0.30%–0.037%) sulfur, which involves significant differences in the elemental composition of the microalga depending on the composition of the culture medium. In those bioprocesses where there is a higher concentration of OIW, a lower % N is noted. The highest increase in the % C and % H in the microorganism linked to a reduction in the % N, has been observed in BP1 and BP2, bioprocesses with the biggest percentage of OIW (50%). This behavior is attenuated with increasing initial inoculum concentration (BP2). While, the higher % N has been found in the bioprocesses enriched with the R‐L culture medium, BP12, BP13, and BP14. The BP14 corresponds to a reference state in which no proportion of wastewater has been considered. (50% of R‐L and 50% double distillate water).

**TABLE 4:** Elemental analysis of bioprocesses (± standard deviation).

Biopr.	% N	% C	% H	% S	N/C
BP1	1.91 ± 0.02	51.18 ± 0.03	7.48 ± 0.08	0.01 ± 0.00	0.037 ± 0.001
BP2	2.62 ± 0.07	49.36 ± 0.04	6.96 ± 0.06	0.26 ± 0.02	0.053 ± 0.002
BP3	3.55 ± 0.03	40.57 ± 0.02	5.66 ± 0.06	0.02 ± 0.00	0.087 ± 0.001
BP4	4.14 ± 0.05	45.85 ± 0.05	6.64 ± 0.07	0.29 ± 0.00	0.042 ± 0.001
BP5	4.13 ± 0.03	42.99 ± 0.06	6.40 ± 0.07	0.02 ± 0.00	0.096 ± 0.001
BP6	3.95 ± 0.05	50.20 ± 0.05	7.63 ± 0.08	0.30 ± 0.02	0.058 ± 0.002
BP7	3.14 ± 0.06	42.19 ± 0.06	6.41 ± 0.05	0.17 ± 0.02	0.074 ± 0.002
BP9	6.72 ± 0.07	34.26 ± 0.02	5.41 ± 0.06	0.18 ± 0.02	0.196 ± 0.003
BP10	4.11 ± 0.05	32.33 ± 0.05	5.12 ± 0.05	0.19 ± 0.02	0.127 ± 0.002
BP12	7.91 ± 0.06	38.97 ± 0.04	5.38 ± 0.06	0.13 ± 0.02	0.203 ± 0.002
BP13	5.63 ± 0.05	46.52 ± 0.06	6.54 ± 0.07	0.12 ± 0.02	0.121 ± 0.001
BP14	6.04 ± 0.02	45.26 ± 0.06	6.81 ± 0.00	0.33 ± 0.03	0.133 ± 0.000

The relationship between the composition of the culture medium and the elemental composition of the microorganism has also been studied *Chlorella sorokiniana* [55] as well as the relationship between the microalgal N and the P composition of medium [56]. Also, *Chlorella* and *Scenedesmus* showed be able to adjust the internal concentration of N and P in the biomass according to the concentration of N and P in the wastewater medium [57].

### 3.3 *Neochloris oleoabundans* Growth

The maximum specific growth rate in the exponential phase has been calculated by least squares fitting to the Equation (2).

(2) $$\mathrm{Ln}\left(x/x_{o}\right)=\mu_{m}t+a$$

where “*µ_m_*” is the slope of the line and corresponds to the maximum specific growth rate and “*a*” is the intercept. The identification of the linear phase has been carried out from the representation in linear coordinates of the biomass concentration values *x* (g dm_−3_) versus time, *t* (*h*). This deceleration in growth is located immediately after the exponential phase as seen in Figure 2. The adjustment of *x* versus *t* was done through Equation (3).

(3) $$x=P_{b}t+b$$

where “*P_b_*” is the slope of the line and corresponds to the volumetric biomass productivity and “*b*” is the intercept. The adjustment of the experimental results to this mathematical model (Equations 2 and 3) has made it possible to determine the different kinetic parameters (Table 5) *µ_m_* and *P_b_* from the biomass concentration and Ln(*x*/*x*_0_) values plotted against time for all crops.

**TABLE 5:** Values of maximum specific growth rate, volumetric productivities and maximum biomass concentration (± Standard Deviation).

Inoculum conc.	1.1 g dm^−3^				0.3 g dm^−3^		
Exp.	*µ_m_*, h*^–^* ^1^	*P_b_*, g dm*^–^* ^3^ h*^–^* ^1^	X_max_ g dm*^–^* ^3^	Code exp.	*µ_m_*, h*^–^* ^1^	*P_b_*, g dm*^–^* ^3^ h*^–^* ^1^	X_max_ g dm*^–^* ^3^
BP1	0.022 ± 0.003	0.90 10*^–^* ^3^ ± 0.006	1.27 ± 0.03	BP2	0.038 ± 0.007	0.86 10*^–^* ^3^ ± 0.008	1.31 ± 0.07
BP3	0.012 ± 0.002	1.80 10*^–^* ^3^ ± 0.009	0.56 ± 0.04	BP4	0.019 ± 0.008	1.07 10*^–^* ^3^ ± 0.010	0.31 ± 0.06
BP5	0.013 ± 0.004	2.70 10*^–^* ^3^ ± 0.014	1.04 ± 0.03	BP6	0.012 ± 0.005	2.25 10*^–^* ^3^ ± 0.009	1.14 ± 0.02
BP7	0.049 ± 0.012	1.0 10*^–^* ^3^ ± 0.005	1.53 ± 0.05				
BP8	0.036 ± 0.013	1.60 10*^–^* ^3^ ± 0.008	0.68 ± 0.02				
BP9	0.007 ± 0.001	1.80 10*^–^* ^3^ ± 0.007	0.85 ± 0.02				
BP10	0.035 ± 0.011	0.90 10*^–^* ^3^ ± 0.005	1.00 ± 0.04	BP11	0.032 ± 0.008	0.40 10*^–^* ^3^ ± 0.003	0.83 ± 0.02
BP12	0.082 ± 0.025	3.00 10*^–^* ^3^ ± 0.022	2.98 ± 0.01	BP13	0.065 ± 0.015	1.70 10*^–^* ^3^ ± 0.008	1.93 ± 0.05
BP14	0.020 ± 0.007	2.90 10*^–^* ^3^ ± 0.018	2.14 ± 0.04				

Six bioprocesses, including one control (BP 14 with 50% of R‐L and 50% double distillate water), have been considered in Figure 2 to show the growth curves of *N. oleoabundans*, allowing the exponential phase to be identified.

In general, a short duration (±20 h) of lag phase was detected in all experiments, despite the initial concentration of inoculum (high or low). This phase took longer (±55 h) for the growth medium containing a relatively higher amount of OIW in BP1, 2, and 7, due to the presence of high organic matter, as indicated by high turbidity = 935 NTU and BOD_5_ = 43,000 mg O_2_dm^−3^. The change in composition aimed at 10% OIW and 50% OLW (BP8, BP3, and BP4) shortened the lag period. Taking into account the higher phosphate, nitrite, and nitrate content present in OIW, this behavior is in line with the study in which, *N. oleoabundans* grew exponentially during (±92 h) and consumed nitrate and phosphorus (in the form of orthophosphate) ions available in the medium during this growth phase [58].

Figure 2C shows the kinetic parameters of BP1, constituted by a percentage of 50% of OIW, which represents the most problematic type of OMW with its high values of phenolic compounds, nitrogen, and orthophosphates (Table 2). The highest values of *µ_m_* = 0.022 h^−1^ and *P_b_* = 0.9 mg dm^−3^ h^−1^ are reached with an initial OIW concentration of 50%. These lower values of *µ_m_* and *P_b_* could be justified by the major content of phenolic compounds in OIW. The X_max_ was reached in these conditions, increasing with the lower initial inoculum concentration. The values of *µ_m_* were lower compared to when the nutrient medium consisted of UWW, and volumetric productivities were lower than those made up of the same proportion of OLW (BP3) or UWW (BP5). These lower *µ_m_* values in BP3 and BP5 compared to those in BP1 could be justified considering the high initial concentrations of nutrients such as nitrates, nitrites, orthophosphates, and sulfates contributed by OIW (Table 2). The major proportion of UWW led to higher *P_b_*.

For the growth of *Chlorella vulgaris* in bubble column photobioreactors with the gelatin industry wastewater was prepared with different dilutions and supplemented with medium, the results achieved were *µ_m_* = 0.014 h^−1^ and *P_b_* = 5.87 10^−3^ dm^−3^ h^−1^ [59], with minor polyphenol content and lower BOD_5_ and COD.

In the case of BP 10 (Figure 2E) (10% OIW, 10% OLW, and 30% UWW), the average values X_max_, *µ_m_*, and *P_b_* were 1.0 g dm^−3^, 0.035 h^−1^ and 0.9 mg dm^−3^ h^−1^, respectively. This mixture of the wastewater confers higher values than those obtained working with *Chlorella pyrenoidosa* using 20% OMW [60]. Comparing the results of both works, *N. oleoabundans* seem to have more ability than *C. pyrenoidosa*, under similar growing conditions, in consuming nitrogen and phosphorus, the two essential components for algae growth, present in higher proportions in OIW.

With *S. obliquus*, the best behavior of the microorganism corresponded to a maximum specific growth rate (*µ_m_*) of 0.074 h^−1^, a biomass productivity (*P_b_*) of 4 mg dm^−3^ h^−1^, and net biomass generation of 0.28 g dm^−3^ was reached to culture medium containing 25% (v/v) UWW with a 5% (v/v) of OMW [18]. The highest *P_b_* value obtained with *N. oleoabundans* was 2 mg dm^−3^ h^−1^, combining the three wastewaters without R‐L medium in BP 9 (10% OIW, 10% OLW, and 50% UWW).

It is observed in BP 12 (Figure 2B), (10% of OIW, OLW, and UWW + 50% R‐L) that the highest concentration of biomass formed in the R‐L medium at 50% enriched by OMW and UWW was 2.98 g dm^−3^. It can be observed that the maximum experimental values of *µ_m_* and *P_b_* were reached after a short lag phase are 0.0821 *µ_m_* h^−1^ and *P_b_* of 3 10^−3^ g dm^−3^ h^−1^. From the results obtained, the average values of maximum specific growth rate and volumetric productivity of biomass are the highest, and then both parameters began to decrease. The presence of OMW and UWW resulted in a 22% increase in the growth of the microorganism compared to BP14 (without wastewater). At the same time, this increased growth was 65% higher than when the initial inoculum concentration was lower (BP13). This enrichment with OMW and UWW leads to high consumption of ions, especially nitrogen derivatives (nitrate and nitrite), as well as phosphate and sulfate.

When 50% R‐L medium is present in the BP12 and B13 mixtures the highest *µ_m_* and *P_b_* values were found for the highest initial inoculum concentration, being the *P_b_* values obtained under these conditions comparable to those obtained without R‐L medium, but with 50% UWW (BP 5 and BP6).

Other factors, such as a limited availability of CO_2_ [61] and light [62] can be influential in the cultures. In this work, CO_2_ was supplied through aeration of the culture medium at 0.018 m^3^ h^−1^ and the incident intensity of the lighting was also constant in all experiments and equal to 359 µE m^−2^ s^−1^. However, due to the coloration of the medium (oil wash waters have a dark color compared to the other types of waters), the light attenuation was higher in the culture medium containing a higher percentage of OIW. This could explain the decrease in *P_b_* with increasing concentration of OIW in the culture medium. Apart from these factors, other, such as the presence of fatty matter, organic acids, pesticide residues, and phenolic compounds in the composition of OIW are known to damage and inhibit the growth of microalgae. [1]. The combination of these three wastewater fractions results, due to its composition, in a suitable nutrient medium for the development of *N. oleoabundans* while contaminant OMW and UWW could be removed from the OMW treatment circuit.

### 3.4 Recovery of Bioproducts from the Post‐Treated Biomass

Starting from the initial composition of *N. oleoabundans* [63, 64], the final composition in lipids, carbohydrates, and proteins of the *N. oleoabundans* harvested at the end of the cultures can be observed in Figure 3. No variation was detected in the total pigments (0.05 < % Pig. < 1 ± 0.05%). Meanwhile, the percentage of crude protein content changes when dealing with OIW (12.00 ± 0.1%) compared to OLW (22.20 ± 0.1%) and UWW (25.80 ± 0.1%). This percentage may change slightly depending on the initial inoculum concentration in the case of 50% OIW and 50% OLW and significantly in the case of 50% UWW. Nonetheless, the presence of R‐L at 50% rises the protein amount (49.45 ± 0.1%) and (37.78 ± 0.1%) in BP12 and BP14, respectively. Meanwhile, total lipid content raised with increasing % OIW in culture medium (50.96 ± 1.00%) and remained constant in medium containing 50% OLW and UWW (29.00 ± 2.00%). A stable behavior was observed for carbohydrate content, but a decrease was noted up to ±29.00%, with the increasing culture having 50% R‐L.

#### 3.4.1 Pigments

In most bioprocesses, the highest percentage of pigments is reached in the initial phase of the experiment, usually between 24 and 48 h. As the experiment progresses, this value generally decreases in all bioprocesses. Generally, the decrease starts at the beginning of the culture, although a slight increase can be observed during the exponential phase before the transition to the deceleration and stationary phase. The variations in mg dm^−3^ of total chlorophyll, without considering BP14 (10.17 mg dm^–^^3^), were: BP6 (7.13 mg dm^–^^3^) > BP10 (6.17 mg dm^−3^) > BP4 (3.29 mg dm^−3^) > BP2 (2.90 mg dm^−3^), similar behavior was observed for carotenoid values. That is, 50% UWW > (10% OIW + 10% OLW + 30% UWW) > 50% OLW > 50% OIW. The crucial role of nitrogen in pigment synthesis, especially in the production of carotenoids in microalgae was found [65]. Results, in this study, seems to indicate that the presence of OIW does not favor pigment development, despite its high nitrogen content in the form of nitrites and nitrates. The negative effect of the high percentage values of phenolic compounds, COD and BOD_5_ in OIW seems to have a larger influence on pigment development. The mixture of the three types of wastewater (BP10), seems to work better with regard to pigment development than when only one type of OMW is added.

The highest pigment percentage values achieved were 1.83% (1.53% for total chlorophyll and 0.30% for total carotenoids), referred to as cell dry weight, corresponding to 3294 *µ*g dm^–^^3^ total chlorophyll and 656 *µ*g dm^–^^3^ carotenoids, respectively, for BP4 with a 50% of OLW. These results are higher than those found working with *Scenedesmus sp*. and bacterial inoculation during the start‐up of paper pulp effluent bioreactors. In their study, the highest total chlorophyll and carotenoid concentrations measured were 880.45 ± 368.20 *µ*g dm^–^^3^ and 419.14 ± 102.39 *µ*g dm^–^^3^, respectively [66]. Working with *N. oleoabundans* by chicken manure waste as feedstock, the values 0.1% of total pigments was not exceeded [31]. Milner et al. found the total chlorophyll content in *C. vulgaris* is between 0.01% and 6.0% depending on the culture conditions, emphasizing that the operating conditions, especially the nutrient concentration, has a significantly influence at the pigment production and the biochemical composition of the microalgae [54].

#### 3.4.2 Proteins

The percentage of P_crude_ referred to cell dry weight using OIW varied between 12% and 16.5%, which was lower than when of OLW and UWW were used, where the percentage varies between 22% and 26% (Figure 3).

The highest values for protein productivity were found in BP12 (0.036 g dm^−3^ day^−1^) >BP14 (0.026 g dm^−3^ day^−1^) >BP5 (0.017 g dm^−3^ day^−1^) >BP13 (0.014 g dm^−3^ day^−1^) (Table 6), corresponding these values expressed in % P_crude_ to 49.5, 37.8, 25.2, and 35.2%, respectively (Figure 3). This supposes a bigger % P_crude_ when the three types of wastewater were added to the medium R‐L (10% OLW + 10% OIW + 10% UWW: BP12). Without R‐L medium, the best result has been obtained when there is more UWW in the medium (BP5). Some authors have found a relationship between protein production and high nitrogen concentration provided [67]. In the indicated bioprocesses, the high elemental nitrogen content in the microorganism can be observed (Table 4), as well as the initial total nitrite and nitrate content in the culture medium shown in Table 8. Likewise, a relationship between an increasing in the number of cells and a decrease in protein content leading to a decrease in the nitrogen and phosphate (N/P) ratio has been found [68]. In sum, the algae began to consume nitrogen from the medium to convert it into energy compounds, and, therefore, its concentration would be reduced in the medium. With *C. pyrenoidosa* a 43.7% of proteins were reached in the biomass from the culture medium enriched in OIW [60]. The protein productivities are shown in Table 6.

**TABLE 6:** Productivities (*P_b_*) for bioprocesses.

Biop.	*P_b_*, Lipids (g dm^−3^ d^−1^)	*P_b_*, Proteins (g dm^−3^ d^−1^)	*P_b_*, Carbohydrates (g dm^−3^ d^−1^)
BP1	0.009	0.003	0.008
BP2	0.011	0.003	0.005
BP3	0.013	0.01	0.016
BP4	0.008	0.007	0.009
BP5	0.018	0.017	0.024
BP6	0.017	0.01	0.022
BP7	0.003	0.005	0.014
BP8	0.009	ND	ND
BP9	0.009	0.008	0.022
BP10	0.002	0.006	0.010
BP11	0.002	ND	ND
BP12	0.014	0.036	0.015
BP13	0.007	0.014	0.015
BP14	0.028	0.026	0.008

Abbreviation: ND, not determined.

The evolution of the percentage content of cellular proteins was determined by the colorimetric method of Bradford [42] is shown in Figure 4A. This evolution was interpreted together with cell growth (Figure 4B), in order to understand the relationship between essential nutrient availability, biomass development, and protein accumulation in five different bioprocesses (1, 3, 5, 10, and 14).

Finding that the decrease in percentage protein in relation to the increase in biomass is more accentuated for the BP14 bioprocess (50% R‐L + 50% BDW), the nitrate‐ and orthophosphate‐rich medium generated a highly favorable environment for algal growth. A high initial protein content was observed, followed by a slight decrease, probably due to a metabolic redistribution of resources towards intense cell proliferation. (Figure 4B). This response is consistent with the behavior described in other works in enriched media, where structural biosynthesis is prioritized over protein storage [69, 70].

The variation throughout the experiment in percentage protein was less relevant in BP1, BP3, and BP5, associated with a less accentuated cell growth, due to the initial available nitrogen content as well as to the high organic load and the toxicity associated with phenolic compounds generated an unfavorable environment for cell metabolism, according to results found in studies working with *N. oleoabundans* requiring an adequate and balanced nutritional conditions to maintain optimal rates of protein synthesis and growth [71, 72].

#### 3.4.3 Total Lipids

The high initial fatty matter content of *N. oleoabundans* [27, 73], makes it a suitable microorganism for lipid production. In all bioprocesses, the biomass of *N. oleoabundans* was analyzed to determine the percentage of total lipids at the end of the experiments.

The percentage of total lipids is shown in Figure 3, reaching the highest percentage of lipids in BP2 (51%: 0.65 g dm^−3^), with 50% of OIW. With 50% UWW, corresponding to BP6 31% of lipids were achieved. Also, with 50% OLW in BP3 and BP4, the percentages of 29.6% and 29.5%, respectively, were reached. These values are notably higher than those reported with *N. oleoabundans* under nitrogen‐depleted conditions, finding a lipid content of approximately of 20% [30]. Likewise, working in nitrogen starved conditions a 35%–54% of lipids were found [74]. In a culture medium containing 25% UWW with 5% of OMW, 44.9% of lipids were determined [19].

However, the initial availability of total nitrogen in the synthetic medium of R‐L was 140 mg dm^−3^ [33]. The bioprocesses in which more lipids were produced were those without R‐L medium, in which nitrogen availability decreased, and were performed under nitrogen stress conditions. On this matter, these results are consistent with those obtained in previous studies in which microalgae were cultivated under stress conditions such as high seawater concentration, N/P limitation or high salinity. Under stress conditions, lipids are preferred storage compounds due to their highly reduced state and were packaged in cells for microalgae survival [69, 75]. Other important parameters affects the biomass growth and lipid productivity as the supply of light or a rising of pH from 8 to 10 [76]. Lipids productivities are shown in Table 6

#### 3.4.4 Carbohydrates

Carbohydrates were estimated considering that the sum of % lipids, % proteins, and % carbohydrates must be 90% [43, 44, 45], shown in Figure 3. The remaining 10% corresponded to 4% of nucleic acids [46] and to the percentage of ash measured at the end of bioprocesses that resulted in values around 6%, considering this value as an average value [46].

The larger percentages of carbohydrates (59.7% and 50.2%) were reached in BP7 (50% OIW + 10% OLW + 10% UWW) and BP9 (10% OIW + 10% OLW + 50% UWW) (Figure 3). Carbohydrate productivities are shown in Table 6. As has been argued, the same happens with the accumulation of lipids as with the production of carbohydrates. The carbohydrate content in biomass from low OMW percentages raised due to nutrient deficiency (mainly nitrogen). In this sense, through photosynthesis, microalgae can convert atmospheric CO_2_ together with water and light into organic matter, with carbohydrates being the main products. Excess fixed carbon is commonly stored in carbohydrates, and under stress conditions, these molecular reserves can be used as alternative energy sources for the production of cellular structures [69]. Culture medium containing 25% UWW with 5% of OMW and 72.5% of carbohydrates was determined [19].

Taking into account the initial composition of *N. oleoabundans* cell walls are composed of about 24.3% carbohydrates, 31.5% proteins, 22.2% lipids, and 7.8% inorganic material [70]. The initial situation in which the protein content is predominant, can be addressed in the following way nitrogen depletion conditions to a higher accumulation of lipids and carbohydrates. This way, the final biomass produced could be used in combination with other substrates for the production of biofuels or as a complementary substrate in the anaerobic digester for the production of biogas [10].

### 3.5 Pollutant Component Removal Rate

In order to calculate the reduction of the contaminant load, the following were determined: nitrates, nitrites, sulfates, magnesium, copper, boron, potassium, sodium, and total phosphates (total P) and total phenolic compounds, as well as EC and DO.

#### 3.5.1 Total Phenolic Compounds (TPC) and Chemical Oxygen Demand (COD) Removal

The highest removal rate of phenolic compounds was achieved with *N. oleoabundans* with a value of 65.66% from the culture with BP10 OMW (10% OIW, 10% OLW, and 30% UWW). This percentage of elimination decrease with the augmentation of the volume of OMW especially the OIW, as shown in Table 7.

**TABLE 7:** Removal percentage of total phenolic compounds (TPC) and chemical oxygen demand (COD) with *N. oleoabundans*.

Bioprocess	TPC FD^a^ (mg dm*^–^* ^3^)	TPC LD^b^ (mg dm*^–^* ^3^)	% R^c^ of TPC	COD FD^a^ (mg dm*^–^* ^3^)	COD LD^b^ (mg dm*^–^* ^3^)	% R^c^ of COD
BP1	54.36	35.90	33.96	9630	2520	73.83
BP2	44.40	33.10	25.45	8130	1780	78.11
BP3	2.92	1.20	58.90	1013	270	72.66
BP4	3.30	2.10	36.36	880	230	73.86
BP5	1.90	1.10	42.11	77	22	71.43
BP6	1.50	0.95	36.67	71	20	71.83
BP7	47.10	30.10	36.09	6970	1860	73.31
BP8	11.50	5.10	55.65	2440	570	76.64
BP9	16.90	8.20	51.48	570	133	76.67
BP10	16.60	5.70	65.66	1270	80	93.70
BP11	16.60	6.50	60.84	1260	92	92.70
BP12	11.60	8.40	27.59	2000	180	91.00
BP13	11.60	8.60	24.14	1830	170	90.71
BP 14	0.22	0.1	54.55	82	10	87.80

^a^First day (FD).

^b^Last day (LD).

^c^Removal percentages (% R).

With *C. vulgaris*, *Acutodesmus obliquus*, and *Monoraphidium braunii* phenolic compounds were removed between 7% and 21%. According to Lindner and Pleissner [77], phenolic compounds were removed between 7% and 21% by all the species analyzed (*C. vulgaris*, *A. obliquus*, and *M. braunii*) and could not reduce more than 5% in OMW at 6% (v/v) under light conditions. This illustrates the improved behavior of *N. oleoabundans* when no other carbon source is used (without R‐L medium), demonstrating its capability to use phenol as a carbon source. In another work, it was studied that the carbon source used in combination with the phenolic compound exerted a strong influence on the growth of the culture [78].

The remediation of COD and TPC was particularly relevant because high organic loads and the presence of antioxidant compounds (such as phenolic compounds) are not desirable in wastewater intended to be reused for irrigation or discharged into public water networks. In this sense, other authors have obtained lower COD removal levels using industrial wastewater as culture medium for different microalgae strains. Here, the better removal route restarted with the bioprocess with less concentration of OIW (10%), with 93.70% equivalent to 80 mg dm^−3^ in BP 10. In the case of *C. pyrenoidosa* with OMW 100% where the removal rate was 86% with 187 mg dm^−3^ [60]. Other authors have obtained lower COD removal levels when using OMW as a culture medium for different microalgae strains. Culturing *Spirulina platensis* in OMW, COD removal percentages ranging from 28.8% to 66.9% [79]. *Chlorella zofingiensis* gave rise to 37% COD removal from OMW [80] and with *S. obliquus* a 92.6% [19]. A percentage of removal of COD with *Chlorella* and *Scenedesmus* of 91.7% was reached [81].

Better nutrient removal performance seems to be observed in bioprocesses with larger inoculum concentrations, which is in line with what is found in hyper‐concentrated algae cultures (>1.5 g dm^−3^) showing to be highly effective, accelerating nutrient removal, compared to normal cultures [82].

#### 3.5.2 Ions Removal

In general, a greater decrease was detected in the total content of phosphorus and in nitrogen compounds, which illustrates the needs of algae for these elements, while contributing to the reduction of the concentration of other elements. A reduction in all parameters has been noted (Table 8).

**TABLE 8:** Percentages of ions removed from the wastewater in each of the experiments performed.

	NO_3_ *^–^*			NO_2_ *^–^*			SO_4_^2^ *^–^*			PO_4_^3^ *^–^*		
Biop.	FD^a^ mg dm^−3^	LD^b^ mg dm^−3^	% R^c^	FD^a^ mg dm^−3^	LD^b^ mg dm^−3^	% R^c^	FD^a^ mg dm^−3^	LD^b^ mg dm^−3^	% R^c^	FD^a^ mg dm^−3^	LD^b^ mg dm^−3^	% R^c^
BP1	63.60	13.10	79.40	1.76	0.26	85.23	228	180	21.05	26	8	69.23
BP2	55	15	72.73	1.92	0.62	67.71	200	160	20.00	28	12	57.14
BP3	7.2	1.0	86.11	0.23	0.10	56.14	160	100	37.50	6	2.5	58.33
BP4	7.0	1.2	82.86	0.18	0.10	44.44	170	110	35.29	7.5	3.5	53.53
BP5	3.3	1.0	69.70	2.04	0.14	93.14	180	130	27.78	2.3	1	56.52
BP6	3.7	1.2	67.57	2.29	0.13	94.32	196	155	20.92	3.1	1.5	51.61
BP7	50	13	74.00	1.28	0.48	62.5	210	170	19.05	38	18	52.63
BP8	29	8	72.41	1.40	0.48	65.71	160	140	12.50	28	20	28.57
BP9	33	8	75.76	2.24	0.29	87.05	190	150	21.05	31	10	67.74
BP10	80	11	86.25	1.27	0.44	65.35	150	120	20.00	85	43	49.41
BP11	80	14	82.50	1.27	0.52	59.06	150	120	20.00	85	47	44.71
BP12	83	6	92.77	1.60	0.32	80.00	400	160	60.00	94.5	71	24.87
BP13	83	2	97.59	1.61	0.32	80.12	390	160	58.97	96.5	75	22.28
BP 14	173	15	91.33	0.22	0.08	63.63	280	160	42.85	222	70	68.47

^a^First day (FD).

^b^Last day (LD).

^c^Removal percentages (% R).

**FIGURE 5:**
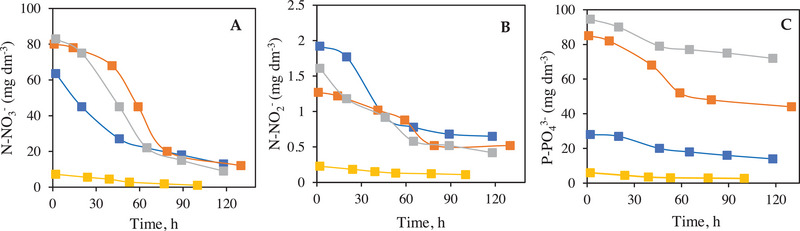
Ions removal by *Neochloris oleoabundans* in BP1 (◼), BP3 (◼) BP10 (◼) and BP12 (◼) on: (A) nitrate removal, (B) nitrite removal, and (C) phosphorus removal.

*N. oleoabundans* is capable of completely depleting N and P from wastewaters containing high concentrations of nitrate and phosphate while achieving high biomass productivity. They are essential nutrients in biomass formation [79]. Figure 5 compares the evolution of four bioprocesses, with respect to the removal of nitrates, nitrites, and orthophosphates, thus for BP10 a net difference of 69 mg dm^−3^ for N‐NO_3_^–^, of 0.83 mg dm^−3^ for N‐NO_2_^–^and 42 mg dm^−3^ for P‐PO_4_^−3^ was removed for each ion. These results are much better than those reported for *C. vulgaris* able to remove 7.7 mg dm^−3^ of P‐PO_4_^−3^ [83]. In this sense, several studies have demonstrated the ability of microalgae to assimilate nitrogen in different forms, with ammonium ions being the preferred source since less energy is required. The presence of phosphorus in the medium is very relevant for the cell growth of microalgae due to its role in metabolism and phosphorylation reactions [84].

*C. vulgaris* has been assigned a removal efficiency of 86% for inorganic N and 70% for inorganic P [85]. On the other hand, when working with *Chlorella sp*. they obtained removals of 75.7%–82.5% for nitrite and 62.5%–74.7% for phosphorus [86]. Using *Chlorella sp*. for the treatment of highly concentrated municipal wastewater, 89.1% for total nitrogen and 80.9% for phosphorus of removal were achieved [87]. With *S. obliquus* a total nitrogen removal (63.5%) was reached [19]. The highest removal rates were found for nitrates (86.25%) in BP10, for nitrites (94.32%) in BP6, without considering the bioprocesses with R‐L medium. In the case of sulfates (37.50%) in BP3 and for orthophosphates (69.23%) in BP1.

Regarding cations removal, a significant removal of magnesium is noted in all 13 bioprocesses and, especially, in bioprocesses 10 and 11, where OMW represents about 65% of removal the medium in BP10, increasing to 87% in presence of R‐L medium, as a sample as shown in Table 9. In another study with *C. vulgaris* had been shown that 69% of the initial Mg^2+^ had been removed evaluating the kinetic model based on a first‐order reversible reaction for the bioaccumulation of Mg^2+^ [88].

**TABLE 9:** Percentage of metals removed calculated from the results obtained in the ICP mass spectrometer.

	Na^+^			Mg^2+^			Cu^2+^			K^+^			B^3+^			Ca^2+^		
Biop.	FD^a^ mg dm^−3^	LD^b^ mg dm^−3^	% R^c^	FD^a^ mg dm^−3^	LD^b^ mg dm^−3^	% R^c^	FD^a^ mg dm^−3^	LD^b^ mg dm^−3^	% R^c^	FD^a^ mg dm^−3^	LD^b^ mg dm^−3^	% R^c^	FD^a^ mg dm^−3^	LD^b^ mg dm^−3^	% R^c^	FD^a^ mg dm^−3^	LD^b^ mg dm^−3^	% R^c^
BP1	327.05	42.36	87.05	29.56	24.71	16.41	0.28	0.26	7.14	687.53	631.98	8.08	1.326	1.058	20.21	122.37	70.53	42.36
BP2	316.87	44.70	85.89	16.63	9.77	41.25	0.18	0.12	33.33	363.23	248.89	31.48	0.427	0.272	36.30	67.57	39.30	41.84
BP3	40.48	26.24	35.18	13.20	9.71	26.45	0.17	0.08	52.94	78.44	55.91	28.72	0.528	0.370	29.92	123.79	96.88	21.74
BP4	22.16	18.60	16.06	8.59	5.73	33.27	0.10	0.07	30.00	47.78	41.08	14.02	0.305	0.243	20.33	83.65	60.78	27.34
BP5	136.45	68.73	49.63	15.06	12.26	18.60	0.06	0.05	16.66	154.22	152.73	0.97	0.668	0.431	35.48	16.25	15.83	2.58
BP6	85.22	57.86	32.10	14.98	11.80	21.22	0.023	0.020	13.04	154.05	136.39	11.46	0.378	0.296	21.69	17.63	16.02	9.13
BP7	570.40	107.80	81.10	—	—	—	0.26	0.21	19.23	816.46	785.30	3.82	—	—	—	131.61	80.18	39.08
BP8	128.24	104.65	18.39	28.96	25.39	12.34	0.21	0.12	42.87	366.73	305.41	16.72	0.826	0.767	7.14	131.82	82.52	37.40
BP9	157.61	149.80	4.96	33.12	24.19	26.98	0.14	0.07	50.00	472.21	431.83	8.55	0.980	0.782	20.20	84.07	24.75	70.56
BP10	175.33	168.40	3.95	18.88	6.61	64.99	0.11	0.07	36.36	—	—	—	0.215	0.203	5.99	21.91	4.72	78.46
BP11	168.90	144.20	14.63	18.84	7.88	58.17	0.11	0.06	45.45	364.70	316.05	13.34	0.245	0.168	31.43	21.35	6.42	69.93
BP12	165.22	114.16	30.90	24.74	12.45	49.66	0.097	0.070	27.83	389.15	272.89	29.87	0.290	0.203	30.00	23.02	5.42	76.45
BP13	153.50	106.57	30.57	22.73	10.92	51.95	0.086	0.050	41.87	474.77	386.20	18.65	0.303	0.192	36.63	31.47	6.13	80.52
BP14	346.15	49.58	85.54	28.04	3.61	86.99	0.065	0.013	79.25	498.67	65.68	86.78	—	—	—	5.07	1.44	71.60

^a^ First day (FD).

^b^ Last day (LD).

^c^ Removal percentages (% R).

The results showed that the presence of Cu^2+^ inhibited the growth of microalgae and, accelerated the formation of photosynthetic pigments, and promotes microalgae to detoxify heavy metals. The % Cu^2+^ removal rates did not exceed 60% at pH 7 [89]. In the case of *Scenedesmus incrassatulus*, 44% of removal was not exceeded [90]. Working with *Oedogonium sp*. a removal rate of 46% was found [91]. In this work, the highest percentage removal rates of Cu^2+^ were in BP3 (53%), BP8 (43%), BP9 (50%), BP11 and BP14 (79%). Removing Cu(II), *N. oleoabundans* exhibited distinctive adsorption behaviors than other metal ions [64].

Removing Na^+^, in BP2 (86%) of removal rate was achieved. For the K^+^ and Ca^2+^ removal, the increasing of stress conditions led to a reduction in the removal rate, which is consistent with what we found working with *S. obliquus* to remove cations (K^+^, Na^+^, Ca^2+^, Mg^2+^) from saline‐alkaline water. Under a salinity stress, the concentration of Na^+^ in *S. obliquus* incremented significantly, while that of K^+^ decreased significantly. The concentrations of Ca^2+^ and Mg^2+^ descended as well [87]. The maximum removal of the cations (29.37 mg for K^+^, 185.85 mg for Na^+^, 23.07 mg for Ca^2+^, and 66.14 mg for Mg^2+^) occurred at salinity 25 mmol dm^−3^. It has also been observed that the reduction rate of B^3+^ is reduced under stress conditions.

The measurements made of EC, DO, in Table 10 shown that the highest reduction rates for these parameters were achieved under conditions of major stress, increasing the quality of the water and its usability. Regarding the rising in DO concentration after microalgae cultivation, photosynthetic activity rather than carbon oxidation has been indicated during heterotrophic growth [79].

**TABLE 10:** Percentage of dissolved oxygen (DO) and electrical conductivity (EC) reduction.

	DO, mg dm^−3^			EC*, µS cm^−1^		
	FD	LD	% Reduction	FD	LD	% Reduction
BP1	9.2	5.98	35.00%	1542	1388	9.99%
BP2	8.21	4.51	45.07%	1334	1245	6.67%
BP3	8.73	5.22	40.21%	752.1	685.9	8.80%
BP4	8.62	4.99	42.11%	698.1	637.2	8.72%
BP5	8.97	5.69	36.57%	995	968	2.71%
BP6	8.5	5.09	40.12%	1008.9	907.5	10.05%
BP7	8.62	5.2	39.68%	2369	2298	3.00%
BP8	8.24	5.37	34.83%	2054	1997	2.78%
BP9	7.97	6.01	24.59%	2086	2011	3.60%
BP10	8.81	5.48	37.80%	2088	2047	1.96%
BP11	8.92	5.43	39.13%	2165	2039	5.82%
BP12	8.98	5.74	36.08%	1987.1	1898	4.48%
BP13	8.61	5.62	34.73%	1922	1871	2.65%
BP14	9.22	5.79	37.20%	ND	ND	ND

Abbreviations: FD, first day; LD, last day.

*Notes:* *EC, FD: liquid sample centrifuged and microfiltrated. *EC, LD: liquid sample only centrifuged.

### 3.6 Removal of Pollutants in Wastewater for Use in Irrigation

The agronomic risks of reclaimed water could damage the soil and irrigated crops due to the presence of chemical substances, which can cause salinity, or specific ionic toxicity. The agronomic risks of recycled water could damage soil and irrigated crops due to the presence of chemicals, which can cause salinity, or specific ionic toxicity [15]. Therefore, these parameters have to be regulated.

The quality of the irrigation water for nil to slight effect has to maintain the following values: pH in the range 6.5–8.4, Electrical conductivity of the water (EC_w_) < 700 µS cm^−1^, (Total Dissolved Solids (TDS) < 450 mg dm^−3^, Na^+^ < 69 mg dm^−3^. Most other crops are relatively unaffected until nitrogen exceeds 30 mg dm^−3^. SO_4_^2–^ must be < 200 mg dm^−3^, Mg^2+^ < 100 mg dm^−3^, Cu^2+^ <0.2 mg dm^−3^ [14, 15].

Table 11 shows a greater percentage of removal of total solids when the proportion of UWW is larger. In relation to the work exposed here, the BP10 and BP11 as mixtures of OLW, OIW, and UWW showed an important decrease of these parameter, remaining below the established limits. At the end of the experiments, values of TDS (Total Dissolved Solids) values achieved were 7.12 mg dm^−3^ for BP1, 0.60 mg dm^−3^ for BP3, and 0.20 mg dm^−3^ for BP5.

**TABLE 11:** Percentage of Total Solids Removal.

Bioprocess	% total solids removal
*BP 1	25.245 ± 0.003
*BP 3	37.505 ± 0.007
*BP 5	40.500 ±0.003

*Note:* *BP1: 50% OIW + 50% BDW. *BP3: 50% OLW + 50% BDW. *BP5: 50% UWW + 50% BDW.

The treated water obtained after the bioprocesses, has reduced the values of those parameters that would prevent its use for agricultural irrigation, such as the content of polyphenols, COD, and total solids. Likewise, it has reduced the presence of ions such as NO^2–^ or others that would contribute salinity, such as Na^+^, and maintains in range others such as B^3+^, whose tolerance depends on the type of crop for which the water is intended.

Boron is essential for the normal growth of all plants, but the amount required is small (0.2 mg dm^−3^). If it exceeds a certain tolerance level in individual plants, boron may be toxic (1 to 2 mg dm^−3^). Boron is poorly absorbed by soils. Irrigation water containing more than 1.0 ppm boron may cause toxicity in boron‐sensitive plants. In general, for all crops boron concentration is satisfactory in terms of tolerance, although between 0.5 and 1.0 mg dm^−3^ is satisfactory for most crops, indeed is satisfactory for semi‐tolerant between 1.0 and 2.0 mg dm^−3^, but is satisfactory for tolerant crops only between 2.0 and 4.0 mg dm^−3^ [92, 93]. After bioprocesses, values of B^3+^ are below 0.5 mg dm^−3^ except in BP1, BP8, and BP9, which are 1.058, 0.767, and 0.782, respectively. It would be possible to state that in the presence of OIW, major concentrations of boron have been found. These concentrations could only be satisfactory for semi‐tolerant crops.

As regards the effect of sodicity, leads to reduced downward movement of water into and through the soil, and the roots of actively growing plants may not receive enough water even though water accumulates on the soil surface after irrigation [94]. It can also cause foliar lesions [95]. The sodium content is very low with the sole presence of OLW (BP3 and BP4), which does not cause any harm to plants. In the rest of the bioprocesses, the Na^+^ content is located below 230 mg dm^−3^, which could be used for the irrigation of certain types of crops.

The reuse of reclaimed water for agricultural irrigation is the oldest and most widespread application today. Currently, the amount of reclaimed water used in agriculture worldwide is ten times greater than the demand for any other use. In Spain, it accounts for around 68% of total water demand and 69% worldwide. Although it is a large quantity, of the three main groups of water use, it is the one with the lowest quality requirements. This water is supplied directly from reservoirs or rivers to irrigation ponds or via agricultural canals to the distribution points of the various farms. In addition, numerous scientific studies have shown that the use of water is not only a cost‐effective source of water but also leads to fertilization of the soil, which arises crop yields and reduces the use of chemical fertilizers.

## 4 Concluding Remarks

Given the experimental results obtained in this work, it can be concluded that *N. oleoabundans* has the capacity to grow in both wastewaters, OWM, and UWW. Regarding the bio‐products recovery, it can be observed that during the course of each experiment, the values of the protein concentration are different. Concluding that the highest percentage of crude proteins without contemplating the BP´s containing a synthetic R‐L medium, was recorded by 25%, with a larger proportion of UWW from secondary treatment (UWW). The highest total lipid content reached was of 51% of dry biomass with a higher proportion of oil wash wastewater (OIW). The elemental composition of the biomass collected in all experiments presented the highest nitrogen content 6.72%, while the highest carbon ratio of 51.2% was obtained with a major proportion of oil wash wastewater (OIW).

In all bioprocesses, a reduction in the parameters measured was achieved. The percentage of removal of contaminants reached by *N. oleoabundans*, after the bioprocess was in nitrite 94.0%, in nitrate 97.6%, in COD 93.7%, and in total phenolic compound 65.7%. The bigger reduction observed in orthophosphate content was 69.2%.

To reduce the % Na^+^ and generate more lipids, a higher proportion of OIW has been favorable, and in general, the use of the three types of water has increased the production of lipids and carbohydrates. The contribution of OMW has led to an improvement in microorganism growth, compared to the bioprocesses with R‐L medium. A bigger growth of *N. oleoabundans* seems to be observed at a greater inoculum concentration, when R‐L medium is present in the mixture. Nevertheless, different combinations of water proportions could be used depending of the particular goal to achieve.

In general, the composition of the mixtures used in bioprocesses increased the lipid and carbohydrate content of the microorganism, which favors the production of biomass that can be used for biofuel production. It can reduce the pollutant load, minimizing parameters that cause it. The results obtained, for the different mixed proportions, make them suitable for certain uses, depending on the specific requirements. With the proportions used, large volumes of polluting waters (OIW, OLW, and UWW) can be reused.

This wastewater has the potential to provide essential nutrients, such as nitrogen and phosphorus, serving as a low‐cost substrate for OMW. Therefore, not only a harnessing of the microalgal biomass, transforming the growth of the microorganism into bioproducts has been achieved, but in parallel the quality parameters of the wastewater have improved, being able to be used for irrigation or returned to natural watercourses. The combination of various urban and industrial wastewaters (OIW and OLW) enables the formulation of an optimal culture medium for microalgae growth, enhancing both economic viability, particularly for biofuel production, and the efficiency of wastewater bioremediation for irrigation. These processes, facilitate wastewater recovery and recirculation, as consequence, could be used simultaneously to increase the circular economy in the olive sector and minimize environmental pollution, reducing the load of pollutants in the environment.

## Nomenclature

 

BOD_5_
: biological oxygen demand (g O_2_ dm^−3^)
COD: chemical oxygen demand (g dm^−3^)
DO: dissolved oxygen in water (mg dm^−3^)
EC: electric conductivity (µS cm^−1^)
EC_w_
: electric conductivity of the water (µS cm^−1^)
OMW: olive mill wastewaters, in general
OIW: olive mill wastewater from olive oil extraction that operates with two outlets decanter
UWW: urban wastewater from secondary treatment
OLW: wastewater from olives washing machines
WOP: wet olive pomace
*P_b_*
: volumetric biomass productivity (g dm^−3^ h^−1^)
R‐L: Rodríguez‐López mineral culture medium
x: biomass concentration (g dm^−3^)
x_0_
: initial biomass concentration (g dm^−3^)
*µ_m_*
: maximum specific growth rate (h^−1^)
A_550_
: absorbance at 550 nm
P_Crude_
: crude protein
TDS: total dissolved solids

## Conflicts of Interest

The authors declare no conflicts of interest.

## Data Availability

The data that support the findings of this study are available from the corresponding author upon reasonable request.
